# Ceftriaxone-Induced Acute Encephalopathy in a Peritoneal Dialysis Patient

**DOI:** 10.1155/2014/108185

**Published:** 2014-12-07

**Authors:** Sami Safadi, Michael Mao, John J. Dillon

**Affiliations:** Division of Nephrology and Hypertension, Mayo Clinic, 200 1st Street SW, Rochester, MN 55905, USA

## Abstract

Encephalopathy is a rare side effect of third and fourth generation cephalosporins. Renal failure and preexisting neurological disease are notable risk factors. Recognition is important as discontinuing the offending agent usually resolves symptoms. We present a case of acute encephalopathy in a patient with end stage renal disease (ESRD) treated with peritoneal dialysis (PD) who received intravenous ceftriaxone for peritonitis. This case illustrates the potential severe neurologic effects of cephalosporins, which are recommended by international guidelines as first-line antimicrobial therapy for spontaneous bacterial peritonitis.

## 1. Introduction

Encephalopathy is a rare side effect of cephalosporins. It is more common in fourth generation cephalosporins [1, 2]. However, it has been reported in third generation cephalosporins as well [3]. Predisposing risk factors include kidney impairment and preexisting neurological disease. We show here a case of acute reversible encephalopathy in a patient with end stage renal disease who received intravenous ceftriaxone for peritonitis. The case highlights key issues with diagnosing and managing this side effect of cephalosporins.

## 2. Case Presentation

A 37-year-old female, receiving PD for ESRD due to lupus nephritis, presented with* Yersinia enterocolitica* peritonitis. She has been on PD for 4 years. She performed continuous cycling peritoneal dialysis nightly. Her PD prescription included 6 exchanges, 1700 mL each, over 9 hours, and a one-day dwell of 500 mL. She used 1.5% dextrose for all exchanges. Her dry weight was 45 kg. Her medication regimen consisted of a metoprolol, sevelamer carbonate, darbepoetin, mycophenolate mofetil, and a low dose prednisone. On exam, her abdomen was soft and nontender. PD exit site was clean with no discharge or erythema.

**Figure 1:**
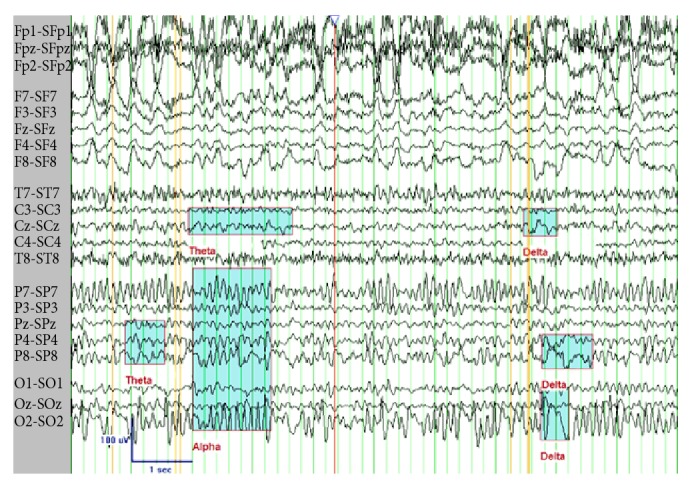
Awake EEG using transverse Laplacian montage, showing normal 9 Hz alpha rhythm in the posterior head regions, as well as abnormal 5-6 Hz theta and 2–4 Hz delta slowing seen diffusely but most prominent in the right posterior temporal (P8), parietal (P4), and occipital (O2) head regions.

She was initially treated with intraperitoneal ceftazidime (125 mL/L). Two days later, she was hospitalized for possible sepsis as her PD cultures grew* Yersinia enterocolitica*. She was switched to intravenous ceftriaxone (2 grams daily). PD was continued. She defervesced quickly and remained hemodynamically stable and her blood cultures were negative. After 3 days of intravenous ceftriaxone, the patient developed agitation, paranoia, and visual hallucinations. The neurological examination was nonfocal. An EEG (Figure 1) showed background moderate diffuse nonspecific slowing without epileptogenic activity. An MRI showed cerebral and cerebellar volume loss but no focal findings to account for the patient's symptoms. She was not receiving any pain medications or centrally acting agents at the time. Furthermore, hallucinations occurred a few hours after ceftriaxone infusions, so ceftriaxone was suspected. The symptoms resolved completely, within 36 hours, after ceftriaxone was switched to ciprofloxacin.

## 3. Discussion

Adverse drug reactions (ADRs) are a serious problem in modern health care. A meta-analysis of prospective studies from US hospitals revealed an overall incidence of 6.7% for serious ADRs (requiring hospitalization or resulting in permanent disability or death), 0.32% for fatal ADRs, and 15.1% for all-severity ADRs [4]. This study further illustrated the clinical impact by showing that, in 1994, 106,000 deaths were estimated to be caused by ADRs in the United States, placing fatal ADRs as the fifth leading cause of death. The majority of ADRs (76.2%) were dose-dependent reactions [4].

Among the many potential reactions to drugs, delirium is a well-known side effect that can lead to increased hospital duration and patient morbidity and mortality. Elderly hospitalized patients, who often may already have multiple risk factors for delirium, such as fluid, electrolyte, metabolic, and sensory/environmental disturbances, are at particularly increased risk when drug toxicity is added to the mix [5]. Chronic kidney disease (CKD) patients share many of the same identified risks for delirium and encephalopathy as elderly hospitalized patients. In addition, they are at a higher risk for fluid, electrolyte, and metabolic abnormalities. Furthermore, the pharmacokinetic and pharmacodynamic parameters of many drugs are altered in CKD [6, 7]. Unfortunately, drug dosing in CKD is often not performed correctly, with inadequate adjustments for renal impairment in 19–67% of hospitalized patients and 42% of long-term care residents [8, 9]. In the ambulatory setting, among nondialysis CKD patients receiving antibiotics, 66% of medications were dosed erroneously [10]. A retrospective observational study showed that necessary renal dose adjustments were not performed for 81% of medications [6]. Among patients with baseline risk factors for delirium and drug toxicity, multiple acute concomitant contributing insults, and inadequate renal drug adjustments, it is no surprise that delirium is a prevalent complication with medications such as narcotics, benzodiazepines, anticholinergic drugs, methyldopa, and nonsteroidal anti-inflammatory agents [5].

This case report however highlights an unusual etiology of delirium and encephalopathy from a common third generation cephalosporin that is currently recommended in the International Society for Peritoneal Dialysis (ISPD) guidelines as a first-line empiric antibiotic for peritonitis, with treatment duration potentially lasting for 14–21 days [11]. It is further worth noting that antimicrobials constituted the majority of the 81% of medications that were not adequately adjusted for CKD in the retrospective, observational study above [6]. Hence, it is worthwhile to raise awareness of the potential for cephalosporin neurotoxicity, especially in this vulnerable population, as it is a dose-dependent effect and rapid recognition of the offending agent would allow rapid recovery [12]. CNS side effects of cephalosporins have also been described in prior case reports that identified renal failure and prior central nervous disease as important risk factors for cephalosporin encephalopathy, often occurring with generalized triphasic waves on EEG [1, 3, 13].

There have been five peritoneal dialysis patients reported in the literature with cefepime-associated encephalopathy [2]. Ceftriaxone-associated encephalopathy has been previously described with CKD [3]; however, to our knowledge, ceftriaxone-associated encephalopathy in peritoneal dialysis has not been described.

Ceftriaxone has a long elimination half-life ranging between 5.8 and 8.7 hours. Between 33 and 67 percent of a dose is excreted in the urine as unchanged drug, and the remainder is secreted in the bile and ultimately is found in the feces as microbiologically inactive compounds [14]. Minimal alterations in the pharmacokinetics of ceftriaxone were, however, observed in patients with renal impairment compared with healthy adult subjects. The average elimination half-life of ceftriaxone is 14.7 hours in patients on hemodialysis and 15.7 hours in patients with severe renal impairment (creatinine clearance 5 to 15 mL/min) [15]. Thus, according to the manufacturer, no adjustment in ceftriaxone dose is needed in renal impairment. They recommend, however, not to exceed 2 grams per day with combined renal and hepatic impairment.

There is a paucity of data regarding the pharmacokinetics of ceftriaxone in PD. However, ceftriaxone seems to exhibit similar pharmacokinetics in hemodialysis and PD patients [16]. Overall, the drug is removed poorly by both modalities. In PD patients with peritonitis, intraperitoneal ceftriaxone administration is preferred as it provides higher concentrations in the peritoneal space. Systemic absorption through the peritoneal membrane is estimated at 40% in patients without peritonitis [17]. The absorption may be higher in peritonitis as ongoing inflammation increases the permeability of the peritoneal membrane. One study looked at the pharmacokinetics of ceftriaxone in CAPD in 8 patients without peritonitis. A single 1.0 g IP dose during a 4-hour dwell time led to serum and dialysate concentrations of ceftriaxone above the minimum inhibitory concentration for susceptible pathogens for 24 hours. The ISPD guidelines recommend a once daily intraperitoneal dose of 1 to 1.5 grams for most cephalosporins in intermittent PD [11]. For continuous PD, half of the dose is given as a loading dose, and the rest is divided over the other exchanges. They, however, do not refer to ceftriaxone specifically.

Our patient had small body size which poses the question whether the drug dose was relatively overestimated for her. However, according to the manufacturer, no weight adjustments are required for ceftriaxone. We also performed a drug interaction analysis using the Micromedex 2.0 drug interaction tool, and this did not reveal any significant interaction with the other medications that our patient was receiving that would alter the pharmacokinetics of ceftriaxone. It is also worth noting that she was not taking any pain medications or centrally acting drugs that can potentially contribute to her altered mental status.

The proposed pathophysiologic mechanisms for cephalosporin CNS effects include inhibition of *γ*-aminobutyric acid (GABA) release from nerve terminals, competitive inhibition of GABA binding to receptor sites, and increased excitatory amino acids [18]. The resulting clinical effects include confusion, hallucinations, cognitive disturbances, delirium, agitation, myoclonus, tremors, convulsions, nonconvulsive status epilepticus, and coma [1, 19]. The temporal pattern of cephalosporin neurotoxicity is an average latency of 1–10 days after drug initiation with regression of all neurological symptoms within 2–7 days after drug cessation [12, 19].

Aside from appropriate renal dosage adjustments and optimization of other mitigating etiologies, there are limited options for reducing the risk for cephalosporin-induced encephalopathy. Genetic studies still have limited applicability in daily clinical practice due to cost and feasibility. Prior studies have identified age, gender, number of medications, alcohol intake, comorbidities, and factors that alter drug distribution or metabolism (heart failure, renal insufficiency, and hepatic insufficiency) as predictors for ADR [20]. More CKD-specific clinical prediction tools for ADR have been attempted using logistic regression formulated risk scores that include age 65 or greater, female sex, conservatively managed ESRD, vascular disease, CRP levels, albumin levels, and eight or greater medications during hospitalization [20]. In daily clinical practice, a high index of suspicion for cephalosporin-induced encephalopathy among vulnerable populations, with immediate cessation of therapy, may still be the most practical approach to limiting this severe ADR.
